# Syrian Medical Association, Hospital at Damascus

**Published:** 1845-04

**Authors:** 


### SYRIAN MEDICAL ASSOCIATION, HOSPITAL AT DAMASCUS.

We have received the following communication from Dr. Thompson, who, as our readers will remember, was last Summer appointed physician to the Dispensary and School of Medicine at Damascus, by the Syrian Association. We at that time gave a full account of the objects of this very laudable charity.

"The climate of Damascus is delightful: the months of July, August, and September, were very hot, yet not oppressively so—a circumstance referable to the dryness of the atmosphere; the thermometer ranged from 98° to 105°; the months of October and November were very agreeable. The periodical rains commenced on the 2nd inst., but continued only for a few days. Though comparatively healthy now, a day seldom passes without forty, fifty, or sixty applications for relief: the largest number of applicants on any day has not exceeded one hundred and eighty. The patients come to the dispensary on three days in each week, the others being devoted to giving out-door relief. Latterly, some cases of midwifery have been attended. I propose, as soon as our funds admit of it, to appoint a female of each sect to the Christian, Jew, and Mohammedan quarters of the city respectively; she will be instructed also in midwifery. I

am sure very many lives would be thus saved yearly. She will see the case first, and then report it, when the patient will be visited and prescribed for. From the instruction thus given to the female practitioner, I hope to break through the few remaining prejudices of the people, and prove to them the importance and superiority of British medical science. I have been here now since July, and have prescribed for three thousand four hundred and fifty patients during that period, of all sects and classes; even the most fanatical come to me for advice, and I have visited the harems of the chief Moslems, the pure descendants of the Prophet. I am now the medical adviser to the Kahia Bey, the governor of Damascus, who has felt so much relieved in British treatment that he has made me a present of a young Arab horse, and has visited me with all his retinue, about twenty-five in number. I attend the Christian and Moslem Leper Asylums, and visit the prison and Lunatic Asylum; I give instructions to the native *Hockums*, and they are quite interested in our modern improvements in surgery. Our electrifying machine astounds the natives, and we have done some good in cases of epilepsy, paralysis, and affections of the retina.

"Dec. 8, 1844.—Since writing the foregoing I have been called upon to visit the Pacha of Damascus, and found the royal patient suffering from catarrh. He is a fine, intelligent old man, of about seventy years of age; was formerly Pacha of Bagdadt. On a former visit, in speaking about the native practitioners, he said it was his creed that no man could successfully practise medicine or surgery who did not know the anatomy of the human frame—a great advance in opinion in such a quarter as this. I have lately prescribed for the Queen Dowager of Persia; her Majesty was veiled very closely, and I was only allowed to see her through the doorway of her bed-room; her tongue and pulse were the only means left me of forming a diagnosis of her condition. However, her Majesty is now well."—*Ibid.*

